# Clinical significance of lower respiratory tract culture within 48 h of admission in patients with viral pneumonia: an observational study

**DOI:** 10.1186/s12890-024-03162-y

**Published:** 2024-07-31

**Authors:** Lu-Lu Chen, Heng Weng

**Affiliations:** Department of Respiratory Diseases, People’ Hospital Affiliated to Fujian University of Traditional Chinese Medicine, No 602, 817 Middle Road, Taijiang District, Fuzhou, 350009 China

**Keywords:** Bacteria, Fungi, Influenza, Pneumonia, Lower respiratory tract specimens

## Abstract

**Background:**

The aim of this retrospective study was to examine the risk factors of positive lower respiratory tract cultures and to investigate whether nosocomial infections are common in patients with positive lower respiratory tract cultures.

**Methods:**

We enrolled 86 patients diagnosed with influenza A-related critical illness who were treated at Fuzhou Pulmonary Hospital of Fujian in China between 1st October 2013 and 31st March 2019. The of admission were used to divide the enrolled patients into two groups. Sputum and bronchoalveolar lavage fluid specimens were collected within 48 h after admission for culture. All samples were cultured immediately after sampling. Nosocomial infections are defined as any symptom or sign of pulmonary infiltration, confirmed by X-ray, after 5 days of admission and positive results from one or more cultures.

**Results:**

The average age of this cohort was (54.13 ± 16.52) years. Based on the culture results, *Staphylococcus aureus* and *Candida albicans* had the highest positive rates (3.40% (3/86) and 20.90% (18/86), respectively). In patients with positive lower respiratory tract cultures, the incidence of nosocomial infection was 73.30% (22/30) five days after admission. However, the incidence of nosocomial infection was lower (42.80%, 24/56) in patients with negative lower respiratory tract cultures. Hemoptysis, systolic pressure at admission, and blood urea nitrogen level at admission were all independent risk factors for positive lower respiratory tract cultures within 48 h of admission.

**Conclusion:**

Our data showed that a significant proportion of patients with pneumonia exhibited co-infections with bacteria or fungi within five days of hospital admission. Hemoptysis, systolic pressure, and blood urea nitrogen levels at admission emerged as the key risk factors. These findings underscore the necessity of closely monitoring patients with influenza infection, particularly for positive bacterial or fungal cultures within the initial 48 h of admission.

## Background

There was a major outbreak of influenza A (H1N1) virus in 2009, resulting in approximately 18,500 deaths worldwide from influenza viral pneumonia [1, 2]. The virus is one of the most common causes of community-acquired pneumonia (CAP), and its detection rate is approximately 5–10% [3, 4]. Many patients infected with the influenza virus are suspected to be co-infected with a bacterium or fungus [5]. According to Martin et al., bacterial co-infection is associated with increased mortality [6]. Other studies revealed a correlation between the influenza A virus and co-infection [7]. The relationship between timely use of antibiotics within 8 h of admission and improvement in pneumonia survival rates has also been reported [8, 9]. Therefore, antibiotic treatment of pneumonia is essential until the presence of a secondary bacterial infection can be ruled out [10].

The bacteriological analysis of specimens taken from the lower respiratory tract presents a significant challenge for doctors [11]. Oropharyngeal flora, such as *Staphylococcus aureus*, *Streptococcus pneumoniae*, and *Candida albicans*, may include potential pathogens and contaminate all expectorated sputum. Therefore, positive cultures from the lower respiratory tract do not necessarily indicate the presence of lower respiratory tract infections [12]. However, lower respiratory tract specimens provide the most direct reflection of a patient’s respiratory tract infection status. Examination of lower respiratory tract specimens can assist in diagnosing related respiratory system diseases and provide reliable evidence for clinical treatment. The clinical significance of positive bacterial or fungal cultures in lower respiratory tract specimens in patients with influenza pneumonia is important. From a clinical perspective, understanding whether the isolated bacteria and fungus are causative agents of lower respiratory tract infections or merely colonizing flora is a significant factor in determining whether antibiotic or antifungal treatment should be administered.

Here, we conducted a retrospective study to assess the risk factors for positive lower respiratory tract cultures and the connection between nosocomial infection and positive lower respiratory tract cultures in patients with influenza A infection. We determined the prevalence of co-infection, identified the causative pathogens, and recommended which patients should be given antibiotics as a precautionary measure upon admission.

## Methods

### Study design and patients

We retrospectively analyzed the medical records of all adult patients with confirmed influenza A-related critical illness who were treated at Fuzhou Pulmonary Hospital of Fujian in China between 1st October 2013 and 31st March 2019. The inclusion criteria were (1) aged ≥ 18 years; (2) tested positive for influenza virus using samples collected within 24 h after admission by the local Centers for Disease Control and Prevention. After admission, respiratory specimens (nasopharyngeal swabs, sputum, or endotracheal aspirates) were collected daily for polymerase chain reaction (PCR) analysis to evaluate the amount of influenza virus RNA [13]; (3) characterized as critically ill, i.e., had a ratio of partial pressure of oxygen in arterial blood (PaO2) to inspired fraction of oxygen (FiO2) less than 300 mmHg (PaO2: FiO2 < 300 mmHg) or required intravenous infusion of an inotropic or vasopressor medication or required mechanical ventilation due to pneumonia complications, septic shock, or multiple organ dysfunction. Exclusion criteria: There was no microbiological culture of lower respiratory tract samples or contamination within 48 h after admission.

### Study definitions

A total of 93 patients diagnosed with the H1N1 or avian influenza virus (H7N9)-related pneumonia were hospitalized from October 2013 to March 2019 at Fuzhou Pulmonary Hospital, Fujian Province in China. Seven patients were excluded from this investigation due to various factors: we failed to collect sputum samples from 4 patients within the allotted time frame; 1 patient was under the age of 18; and 2 patients were transferred to a different hospital.

We evaluated the medical records of the patients and complied the following data: date of hospital and ICU admission, age, gender, date of initial symptoms, laboratory results, radiographic findings, and comorbidities. Standard for lower respiratory tract specimen collection: (1) Sputum samples were taken from all patients before being administered antibiotics or antiviral drugs. If the patient required invasive mechanical ventilation within 24 h of admission, the alveolar lavage fluid sample was obtained. (2) Patients were required to rinse their mouth with clear water 3 times before retaining sputum. Sputum induction by 3–5% sodium chloride atomization was conducted in patients with expectoration issues. (3) All samples of sputum and alveolar lavage fluid were stored in sterile containers.

Within 48 h of hospital admission, cultures were collected from specimens of the lower respiratory tract (trachea), bronchoalveolar lavage, or sputum. All samples were cultured immediately after sampling. After 5 days of hospital admission, any symptom or sign of pulmonary infiltration, excluding those caused by pulmonary embolism, pulmonary edema, and other non-infectious conditions in patients with acute lower respiratory tract infections as confirmed by X-ray and positive results from one or more cultures, was defined as nosocomial infection. On the day of hospital admission, illness severity was measured using the Acute Physiology and Chronic Health Evaluation II (APACHE II) Score, Murray’s Lung Injury Score, the Oxygenation Index (OI), and the Sequential Organ Failure Assessment (SOFA) Score.

Nasopharyngeal swabs were used only for influenza virus detection, not for cultivation. Sputum and bronchoalveolar lavage were used for both influenza virus detection and cultivation. For patients from whom bronchoalveolar lavage or BAL could not be obtained, nasal and throat swabs were used for influenza virus detection, while sputum was used for cultivation.

### Laboratory tests

Laboratory tests were conducted on the day of hospital admission. Every laboratory index was determined in duplicate specimens.

### Statistical analysis

Statistical analysis was performed using SPSS 15.0 Software (IBM Corporation, Armonk, NY, USA). All continuous variables were evaluated for normal distribution using the Kolmogorov–Smirnov test. Categorical variables are presented as a percentage of the total. Student’s t-test and Wilcoxon test were used to compare continuous variables between two groups. The Chi-squared test or Fischer’s exact test were used for categorical data comparisons. Correlations between variables were identified using the Spearman’s or Pearson’s rank correlation coefficient. Parametric data are presented as means ± standard deviation (SD), while nonparametric data are presented as medians and interquartile ranges (IQR). Statistical significance was defined at *P* < 0.05.

## Results

### Patient characteristics

In this study, we enrolled 86 critically ill patients infected with the influenza A virus (H1N1) or avian influenza virus (H7N9); 50 out of 86 patients (58.12%) were over the age of 50 years, and 65 patients (75.58%) were male; the average age was 54.13 ± 16.52 years; 55 out of 86 patients (63.95%) were diagnosed with the H1N1 strain, whereas 31 patients (36.05%) were diagnosed with the H7N9 strain; 40 out of 86 patients (46.51%) had comorbidities, with 30.23% of patients having hypertension and 18.60% of patients having diabetes. Upon hospital admission, the median APACHE II Score, SOFA Score, and Murray’s Lung Injury Score were 16.00 (range, 13.00–21.00), 4.00 (range, 3.00–7.00), and 3.30 (range, 2.70–4.00), respectively. The average OI was 166.66 ± 98.08 mmHg. After hospitalization, 41.86% of patients received mechanical ventilation, and 43.02% of patients received corticosteroids; 72 out of 86 patients (83.72%) were transferred to the intensive care unit (ICU). The median duration of fever before admission was 6 days (range, 4–7); the average number of days with shortness of breath before admission was 4.09 ± 2.95.

All patients enrolled in our study received antiviral therapy. The median period between the onset of symptoms and the initiation of antiviral therapy was 6 days (range, 4–7); 17.40% (15/86) of patients received antiviral therapy within 48 h of the onset of symptoms. The antiviral regimens included 150–300 mg of oseltamivir every day. In all, 91.20% of the patients received antibiotics before admission, and 82 patients (95.30%) were administered empiric antibiotic therapy after admission. The antibiotic regimens were fluoroquinolone monotherapy (35 patients, 40.70%), fluoroquinolones plus beta-lactam (22 patients, 25.60%), carbapenem plus linezolid (17 patients, 19.80%), beta-lactam monotherapy (10 patients, 11.70%), and other combinations (2 patients, 3.40%). Other baseline characteristics are listed in Table 1.

**Table 1:** Overview of the characteristics of all patients

	All patients(*n* = 86)	Culture positive(*n* = 30)	Culture negative(*n* = 56)	*P* value
**Baseline factors**				
Age (years)	54.13 ± 16.52	54.33 ± 19.83	54.02 ± 14.64	0.933^b^
Sex (men), n (%)	65/86(75.58%)	26/30(86.67%)	39/56(69.64%)	0.079^a^
H7N9, n (%)	31/86(36.04%)	10/30(33.33%)	21/56(37.5%)	0.701^a^
H1N1, n (%)	55/86(63.95%)	20/30(63.67%)	35/56(62.5%)	-
APACHE II admission	16.00(13.00–21.00)	19.50(13.00–33.00)	16.00(12.50–18.50)	0.028^c^
SOFA admission	4.00 (3.00–7.00)	5.00(4.00-8.75)	4.00(3.00–7.00)	0.160^c^
Murray’s lung injury score admission	3.30(2.70-4.00)	3.50(3.00–4.00)	3.00(2.50-4.00)	0.097^c^
OI admission(mmHg)	166.66 ± 98.08	153.45 ± 97.86	173.64 ± 98.41	0.382^b^
Heart rate admission (cycles/min)	99.36 ± 18.46	101.97 ± 20.13	97.96 ± 17.54	0.341^b^
Systolic pressure admission (mmHg)	126.56 ± 19.67	132.5 ± 23.36	123.38 ± 16.76	0.040^b^
Diastolic pressure admission (mmHg)	74.37 ± 12.48	75.5 ± 14.84	73.77 ± 11.12	0.543^b^
**Underlying diseases**				
Diabetes	16/86(18.60%)	7/30(23.33%)	9/56(16.07%)	0.409^a^
Hypertension	26/86(30.23%)	11/30(36.66%)	15/56(26.78%)	0.341^a^
COPD	1/86(1.16%)	0/30(0)	1/56(1.78%)	1.000^a^
Cardiac disease	10/86(11.62%)	5/30(16.66%)	5/56(8.92%)	0.475^a^
**Symptom pre-admission**				
Cough	85/86(98.83%)	30/30(100)	55/56(98.21%)	1.000^a^
Expectoration	84/86(97.67%)	30/30(100)	54/56(96.42%)	0.545^a^
Hemoptysis	24/86(27.90%)	12/30(40.00%)	12/56(21.42%)	0.049^a^
Fever	81/85(94.19%)	28/30(96.55%)	53/56(94.64%)	1.000^a^
Dyspnea	71/86(82.56%)	25/30(86.20%)	46/56(82.14%)	0.865^a^
Body aches	19/86(22.10%)	6/30(20.00%)	13/56(23.21%)	0.791^a^
Diarrhea	2/86(2.32%)	0/30(0)	2/56(3.57%)	0.545^a^
**Outcome**				
Length of fever in hospital (days)	2.00(1.00–5.00)	3.00(2.00-7.25)	2.00(0.00–5.00)	0.027^c^
Length of ICU stay (days)	12.00(6.00–20.00)	15.00 (6.00–32.00)	11.00(7.00–17.00)	0.070^c^
Length of hospital stay (days)	19.00(15.00–25.00)	22.00(15.00–37.00)	19.00(14.00–24.00)	0.116^c^
Mechanical ventilation, n (%)	50/86(58.13%)	17/30(56.66%)	33/56(58.92%)	0.839^a^
Nosocomial infection, n (%)	46/86(53.48%)	22/30(73.30%)	24/56(42.8%)	0.007^a^

OI = oxygenation index;

^a^Calculated using the chi-square test

^b^Calculated using Student’s t test

^C^Calculated using the Wilcoxon rank sum test

### Bacterial and fungal cultures from specimens of the lower respiratory tract within 48 h after admission

Bacterial and fungal cultures from lower respiratory tract specimens were obtained within 48 h of hospital admission for 10 and 23 patients, respectively. *Staphylococcus aureus* (3/30, 10.00%), *Stenotrophomonas maltophilia* (2/30, 6.67%), *Flavobacterium indologenes* (1/30, 3.33%), *Klebsiella pneumoniae* (1/30, 3.33%), *Enterobacter cloacae* (1/30, 3.33%), and *Sphingomonas paucimobilis* (1/30, 3.33%) were the bacterial species identified in lower respiratory tract cultures. *Candida albicans* (18/30, 60.00%), *Aspergillus* (7/30, 23.33%), and *Candida tropicalis* (1/30, 3.33%) were the fungal species identified in lower respiratory tract cultures.

According to the culture results, patients were divided into two groups. Positive lower respiratory tract culture groups yielded 30 patients, while negative lower respiratory tract culture groups yielded 56 patients. There were no significant differences in the sex ratio, age, and disease ratio between the positive and negative lower respiratory tract culture groups. Hemoptysis was observed in 12 of 30 patients (40.00%) and 12 out of 56 patients (21.42%) in the positive and negative lower respiratory tract culture groups, respectively. There were no significant differences in the white cell count, lymphocyte count, or C-reactive protein levels between the two groups. The procalcitonin level differed significantly between the positive and negative lower respiratory tract culture groups. The median procalcitonin level in the positive lower respiratory tract culture group was 0.77 ng/ml (IQR, 0.254–1.935 ng/mL) and 0.26 ng/mL (IQR, 0.127–0.776 ng/mL) in the negative lower respiratory tract culture group. Table 2 contains additional information.

**Table 2:** Laboratory indexes of two groups

	Culture positive(*n* = 30)	Culture negative(*n* = 56)	*P* value
W.B.C count (10^9^/L)	5.85(3.66–8.012)	4.93(2.84–6.62)	0.177^c^
Lymphocyte count (10^9^/L)	0.60(0.33–0.93)	0.69(0.46–0.96)	0.174^c^
Platelet count (10^9^/L)	161.40 ± 64.18	154.93 ± 53.83	0.621^b^
C-reactive protein (mg/L)	156.17(58.67-197.23)	97.16(46.04-169.61)	0.113^c^
Procalcitonin (ng/mL)	0.77(0.25–1.93)	0.26(0.12–0.77)	0.015^c^
ALB (g/L)	32.78 ± 4.51	32.29 ± 4.54	0.629^b^
ALT (U/L)	50.50(35.00–86.00)	53.90(31.25-97.00)	0.913^c^
AST (U/L)	60.80(49.25-107.25)	65.00(42.00-108.17)	0.846^c^
ALP (U/L)	84.50(62.00-103.75)	70.00(60.00-96.72)	0.246^c^
BUN (mmol/L)	6.51(5.26–9.81)	4.75(3.85–6.04)	0.000^c^
Cr (umol/L)	81.30(74.20-114.70)	73.85(54.85–91.65)	0.040^c^
LDH(U/L)	568.50(379.25-793.75)	476.00(380.50-767.25)	0.544^c^
CK (U/L)	139.00(69.00-618.25)	175.70(104.75-768.25)	0.220^c^

APTT = activated partial thromboplastin time; ALT = Alanine aminotransferase; AST = aspartate aminotransferase; ALP = alkaline phosphatase; BUN = Blood urea nitrogen; Cr = Creatinine; LDH = lactate dehydrogenase; CK = creatine kinase; CK-MB = reatine kinase isoenzyme;

^b^Calculated using Student’s t test

^C^Calculated using the Wilcoxon rank sum test

### Prognosis

The incidence of nosocomial infection was 73.30% (22/30) inpatients with positive lower respiratory tract cultures 5 days after hospitalization. However, the incidence of nosocomial infection (42.80%, 24/56) was significantly lower (x^2^ = 7.293, *P* = 0.007) in patients with negative lower respiratory tract cultures than in those with positive cultures. Patients with positive lower respiratory tract cultures were hospitalized with persistent fever for significantly longer than those with negative cultures [3 days (IQR, 2–7.25 days) versus 2 days (IQR, 0–5 days), respectively; *P* = 0.027]. There was no significant difference in the duration of the hospital stay between the two groups.

### Logistic regression analysis of factors associated with nosocomial infection 5 days after admission

Five days after admission, the patients in lower respiratory tract culture positive group were divided into two subgroups based on the infection. Among those with positive lower respiratory tract secretion cultures within 48 h, 22 individuals were diagnosed with nosocomial infections five days after admission. Among those with negative lower respiratory tract secretion cultures within 48 h, 24 individuals were diagnosed with nosocomial infections five days after admission. Among the patients with positive lower respiratory tract specimen culture within 48 h after admission, 14 patients had the same microbiological culture-(blood culture or lower respiratory tract specimen culture) diagnosed nosocomial infection 5 days after admission, including 4 patients with *Aspergillus*, 6 patients with *C. albican*s, 2 patients with *S. aureus*, and 1 patient with *Trichosporon asahii*, and 1 patient with filamentous fungi */* yeast-like fungi. Table 3 contains additional information. Next, we conducted univariate and multivariate regression analyses on the occurrence of nosocomial infections five days after admission. The results showed that a positive culture of lower respiratory tract samples within 48 h after admission was not a high-risk factor for the development of nosocomial infections five days after admission (Table 4).

**Table 3:** Clinical analysis of subgroup

	Nosocomial infection(+), Culture (+)	Nosocomial infection (+), Culture (-)		Nosocomial infection (+), Culture (+)	Nosocomial infection (-), Culture (-)	
	*n* = 22	*n* = 24	P	*n* = 22	*n* = 33	p
W.B.C count (10^9^/L)	5.63(3.66–8.67)	4.71(2.90–7.42)	0.281 ^c^	5.63(3.66–8.67)	5.35(2.86–6.52)	0.279 ^c^
Lymphocyte count (10^9^/L)	0.59(0.27–0.98)	0.61(0.38–0.88)	0.582 ^c^	0.59(0.27–0.98)	0.76(0.51–1.21)	0.029 ^c^
Platelet count (10^9^/L)	161.20 ± 71.92	148.4 ± 56.03	0.501 ^b^	161.2 ± 71.92	159.8 ± 51.65	0.932 ^b^
C-reactive protein (mg/L)	162.00(56.44-197.23)	107(49.14–196)	0.480 ^c^	162(56.44-197.23)	97.9(44.26–158.6)	0.059 ^c^
Procalcitonin (ng/mL)	1.13(0.27–2.75)	0.29(0.14–2.19)	0.111 ^c^	1.13(0.27–2.75)	0.26(0.109–0.573)	0.004 ^c^
ALP (U/L)	91.00(76.00–120.00)	66(57–94)	0.036 ^c^	91(76–120)	73(60–98)	0.048 ^c^
BUN (mmol/L)	6.50(5.41–9.68)	4.91(4.14–6.04)	0.013 ^c^	6.5(5.41–9.68)	4.6(3.52–6.04)	0.001 ^c^
Cr (umol/L)	85.00(75.77–117.40)	79.15(58.7-98.25)	0.173 ^c^	85(75.77–117.4)	70.3(53.25–87.85)	0.016 ^c^
LDH(U/L)	651.00(329.00–1257.00)	450(266–833)	0.391 ^c^	651(329–1257)	498(415–731)	0.372 ^c^
CK (U/L)	131(69–637)	193(123–1505)	0.052 ^c^	131(69–637)	150(75–590)	0.594 ^c^

^b^ Calculated using the Wilcoxon rank sum test

^C^Calculated using Student’s t test

**Table 4:** Logistic regression analysis of factors associated with nosocomial infections five days after admission

	Univariate logistic regression analysis			Multivariate logistic regression analysis		
	*P*-value	OR	95%CI	*P*-value	OR	95%CI
Positive culture of lower respiratory tract samples within 48 h after admission	0.008	0.273	0.104–0.717			
Age	0.897					
Cough	1.000					
Hemoptysis	0.533					
Length of fever before admission	0.952					
Shortness of breath before admission	0.029	0.835	0.710–0.982			
OI at admission	0.085					
APACHE II at admission	0.013	1.080	1.016–1.147			
Murray’s lung injury score admission	0.358					
SOFA at admission	0.001	1.420	1.153–1.752	0.01	1.47	1.17–1.845
Intubation after admission	0.000	6.222	2.347–16.498			
Use of hormone after admission	0.002	4.263	1.69-10.757			
Lymphocyte count after admission	0.025	0.299	0.104–0.861			
PCT	0.094					
CRP	0.225					
BUN	0.167					
Cr	0.088					
LDH	0.105					

OR = odds ratio; CI = confidence interval; PCT = procalcitonin; CRP = C-reactive protein; BUN=blood urea nitrogen

### Risk factors for positive lower respiratory tract cultures

Further logistic regression analysis revealed that hemoptysis, the systolic pressure on hospital admission, and the BUN level on hospital admission were all independent risk factors for positive lower respiratory tract cultures obtained within 48 h of hospital admission (Table 5).

**Table 5:** Logistic regression analysis of factors associated with culture positive in lower respiratory tract specimens within 48 h of admission

Variable	OR	95%CI	*P*-value
Hemoptysis	3.911	1.056–14.479	0.041
Length of fever in hospital	1.163	1.017–1.33	0.027
Systolic pressure admission	1.035	1.005–1.066	0.023
BUN	1.228	1.032–1.461	0.021

OR = odds ratio; CI = confidence interval; BUN = blood urea nitrogen

## Discussion

We examined the incidence of nosocomial infection in this population by analyzing lower respiratory tract specimens cultured within 48 h of admission from patients with influenza infection. We also used logistic regression analysis to identify the independent risk factors associated with positive lower respiratory tract cultures from patients with influenza A.

Co-infection is common in patients with influenza infection. Previous studies on patients with influenza pneumonia reported a rate of bacterial co-infection ranging from 20 to 25% of patients [14]. From 2009 to 2015, Martin-Loeches at al., investigated 2,901 patients with influenza infection hospitalized in 148 Spanish ICUs and discovered that 16.6% of them had microbiologically confirmed community-acquired co-infection [6]. Furthermore, specimen cultures from the lower respiratory tract could not be used to categorize patients as being co-infected when they were only colonized. Given the high probability of bacterial co-infection in influenza patients, its association with mortality, and the fact that delaying antimicrobial treatment may result in even higher mortality [15]. In light of our findings, clinicians may be unwilling to tolerate even a low probability of untreated pulmonary co-infection. Empiric antibiotic treatment for co-infection in such patients should be considered until the possibility of co-infection is confidently ruled out. In this study, approximately 91.2% of our patients had received antibiotics prior to admission, and 82 (95.3%) patients received empiric antibiotic therapy after admission.

In this study, we discovered that after 5 days the rate of bacterial infection increased in patients with positive lower respiratory tract cultures, and the length of fever hospitalization was prolonged. The incidence of mortality is highest at 3–7 days after bacterial co-infection [16]. The benefits of antibiotic therapy for viral infection and bacterial co-infection are likely connected to the timing of antibiotic administration, as a correlation between early antibiotic use and enhanced survival has already been demonstrated [17]. Meanwhile, there has been very little experience with using biomarkers as a diagnostic adjunct during influenza pneumonia. Although some biomarkers (particularly procalcitonin) have been linked to bacterial co-infection in this setting, their accuracy is insufficient to determine when antimicrobial treatment should be initiated [18]. In this study, subgroup analysis revealed no significant difference in the results of blood routine, lymphocytes count, C-reactive protein test (CRP) and procalcitonin test (PCT). Gao et al. showed that clinical symptoms and routine laboratory tests cannot distinguish between mixed bacterial infections and simple viral infections [19]. In our investigation, we discovered that the results of microbial culture performed within 48 h of admission were consistent with those of patients diagnosed with nosocomial infection 5 days later. These results suggest that if positive lower respiratory tract culture is obtained within 48 h of admission, regardless of infection or colonization, antibacterial therapy is recommended immediately.

*C. albicans* was reported to be most prevalent in positive lower respiratory tract specimens cultured within 48 h of admission. This result contradicts the findings of previous reports. Van de Veerdonk et al. [20] identified 9 patients infected with a strain of the swine flu (i.e., H1N1) that subsequently developed invasive pulmonary aspergillosis, whose mortality rate is 61%. Wauters et al. revealed that approximately 23% of critically ill patients infected with the H1N1 strain had invasive pulmonary aspergillosis 3 days after being transferred to the ICU [21]. These contradictory findings can be explained by the fact that the high proportion of elderly patients in our study (i.e., > 50% of patients were older than 50 years) and 46.51% of patients had other diseases such as diabetes and/or hypertension. In China, *C. albicans* causes 35% of lower respiratory tract infections in hospitalized elderly patients older than 60 years, and the incidence rate is increasing annually. In addition, nearly two-thirds of the patients included in this study had used second- or third-generation cephalosporins prior to hospitalization.

According to our regression analysis, patients with hemoptysis prior to hospital admission were 3.9 times more likely to have positive lower respiratory tract cultures than those without hemoptysis. It has been has been found that influenza causes significant epithelial cell damage in the lungs [22]. Polymerase component PB1 subunit (PB1-F2) can induce mitochondrial permeabilization and apoptosis with viral infection, which can inadvertently provide nutrition to invading opportunistic bacteria, result in severe cytopathic damage, and destroy the surfactant layer in the lungs [23, 24]. Furthermore, it has been observed that influenza increases the sensitivity of the lower respiratory tract to pathogens, [14] despite the fact that several processes can impact the antibacterial innate immune response. This can render both the upper airways and the lungs susceptible to subsequent bacterial infiltration, thereby leading to increased bacterial load and mortality. These processes include inhibition by type I interferons and alveolar macrophages depletion [25]. However, after infection with influenza A, the virus replicates within epithelial cells of the upper respiratory tract and serves as a receptor for bacteria, which is one of the causes for the high incidence of bacterial infections [26]. Further research is required to define the relevant mechanism.

BUN level seems to be a more sensitive indicator of infection than white blood cell count, C-reactive protein or procalcitonin, according to subgroup analysis. The BUN level is an indicator of acute renal injury, which is common in patients with moderate and severe pneumonia [27]. In patients with pneumonia, dehydration typically leads to an increase in urea by the kidneys, and an elevated BUN level is frequently noted [28]. The findings indicate that infection can damage the kidney. Acute kidney damage could be an early indicator of severe pneumonia complicated with sepsis [29]. Together, these findings suggest that an elevated BUN level, and positive culture results may indicate co-infection. More research is required to confirm this hypothesis.

### Limitations

There were several limitations to this study. To begin, in this study, we analyzed data retrospectively, which limited the ability to infer causality. Second, it was a single-center cohort study with a relatively small sample size. Thus, the data may not be representative of the population under study. Furthermore, the findings were restricted to this population and could not be generalized to other populations. Finally, the small sample size made multivariate analysis less robust.

## Conclusion

In conclusion, we found a high percentage of patients with pneumonia were co-infected with a bacterium or fungus 5 days after hospital admission, as determined by the result of the lower respiratory tract cultures obtained within 48 h of admission. Hemoptysis and an elevated BUN level were the main risk factors. Taken together, these findings may have clinical implications, as patients with positive bacterial or fungal lower respiratory tract cultures obtained within 48 h of admission should be treated immediately and closely monitored. More research is required to confirm these findings.

## Data Availability

All data generated or analysed during this study are included in this article. Further enquiries can be directed to the corresponding author.

## Abbreviations

APTT: Activated partial thromboplastin time
ALT: Alanine aminotransferase
AST: Aspartate aminotransferase
ALP: Alkaline phosphatase
APACHEII: Acute Physiology and Chronic Health Evaluation II
BUN: Blood urea nitrogen
CAP: Community-acquired pneumonia
Cr: Creatinine
CK: Creatine kinase
CK-MB: Creatine kinase isoenzyme
CI: Confidence interval
ICU: Intensive Care Unit
IQR: Interquartile ranges
LDH: Lactate dehydrogenase
OI: Oxygenation Index
OR: Odds ratio
PB1-F2: Polymerase component PB1 subunit
RT–PCR: Probe-based reverse transcriptase–polymerase chain reaction
SOFA: Organ Failure Assessment
SD: Standard deviation
